# Cardiac muscle contracts more efficiently at lower contraction frequencies

**DOI:** 10.1113/EP092367

**Published:** 2025-01-29

**Authors:** Toan Pham, Andrew J. Taberner, June‐Chiew Han

**Affiliations:** ^1^ Auckland Bioengineering Institute University of Auckland Auckland New Zealand; ^2^ Department of Engineering Science University of Auckland Auckland New Zealand

**Keywords:** cardiac energetics, heat, stimulation frequency, ventricular trabeculae

## Abstract

This study investigated how contraction frequency impacts the mechano‐energetics of cardiac muscle performing mechanical work. Left‐ventricular trabeculae were isolated from rat hearts and mounted in our work‐loop calorimeter to assess their function at physiological temperature (37°C) across three stimulation frequencies, 2 Hz, 3.5 Hz and 5 Hz, in a randomised sequence. Each trabecula was subjected to two experimental protocols: work‐loop contractions under a range of afterloads and isometric contractions under a range of muscle lengths. Two contraction protocols allowed the partition of the various components of energy expenditure during cardiac contraction. By simultaneously measuring force–length work and heat output, mechanical efficiency was calculated over a range of afterloads to determine the peak value. Our findings revealed that force production, activation heat (energy associated with Ca^2+^ cycling) and cross‐bridge heat were unaffected by stimulation frequency. Trabeculae produced greater work output per twitch at 2 Hz and 3.5 Hz than at 5 Hz. Positive correlations among work output, shortening extent and mechanical efficiency were detected. From these findings it was concluded that the higher work output at lower frequencies is associated with greater extent of shortening, which correlates to greater mechanical efficiency. This study highlights the mechano‐energetic advantage of ventricular trabeculae in terms of increased work output and energy efficiency gained from operating at lower contraction frequencies, supporting the notion that heart rate reduction produces direct benefits on cardiac energetics.

## Abstract (highlights)

- **What is the central question of this study?**
  Do lower stimulation frequencies affect energy efficiency in ventricular trabeculae under physiological work‐loop conditions?
- **What is the main finding and its importance?**
  There was higher work output per twitch at 2 Hz and 3.5 Hz compared to 5 Hz, attributed to greater shortening extent with the same force production. Total enthalpy remained unchanged and, thus, there was higher mechanical efficiency at lower frequencies. It was concluded that the higher work output drives the increased energy efficiency at lower stimulation frequencies.

## 1 INTRODUCTION

Resting heart rate is closely regulated by the autonomic nervous system to meet the metabolic demands of the body. Physically active individuals typically have a lower resting heart rate compared to sedentary individuals (Fagard, 2003). One study showed that young, healthy individuals with a lower resting heart rate possess higher cardiac energy stores (Fragasso et al., 2011). In patients with cardiovascular diseases, an elevated resting heart rate is commonly observed and is associated with a higher risk of ventricular dysfunction (Tadic et al., 2018; Wang et al., 2017). Since failing hearts operate under the strain of compromised cardiac energetics (Neubauer, 2007), a higher resting heart rate reduces the time for myocardial relaxation and creates an imbalance between oxygen supply and demand. This energetic deficit progressively drives the heart further to irreversible damage, hence exacerbating the progression of heart failure.

Lowering resting heart rate has emerged as a therapeutic strategy for improving outcomes for individuals with congenital heart disease (Ferrari & Fox, 2016) and heart failure (DiFrancesco & Camm, 2004; Dobre et al., 2014; Kjekshus, 1986) by delaying the progression of left ventricular dysfunction and structural remodelling. Pharmacological agents like β‐blockers and ivabradine are widely used to achieve this goal. Heart rate reduction has been shown to decrease myocardial oxygen consumption (Colin et al., 2004), increase the time available for coronary perfusion, and improve cardiac metabolism and left ventricular remodelling in experimental animal models (Ceconi et al., 2009, 2011). Beneficial effects of lowering heart rate with β‐blockers on cardiac energetics were reported in patients with left ventricular dysfunction (Shinke et al., 1999), as demonstrated by the increases in left ventricular ejection fraction and work output per beat with no change in myocardial oxygen consumption per beat and higher energy efficiency. However, other concurrent mechanisms are more likely to contribute to an improvement in energetic function with β‐blocker intervention. For instance, β‐blockers might trigger reflex sympathetic stimulation in response to pacing‐induced hypotension, further augmenting the inotropic state (Asanoi et al., 1994). These drugs may also exhibit inhibitory effects on ionic activities (Van Bogaert et al., 1990). These complex mechanisms may limit the interpretation of the intrinsic role of heart rate reduction on cardiac energetic function.

Isolated muscle preparations provide several advantages in cardiac research by removing the confounding influences of humoral factors, peripheral circulation and side effects of pharmacological interventions. In this study, we investigated the direct impact of contraction frequency on the energetic performance of cardiac muscle in the absence of these confounding influences. We achieved this by studying isolated left ventricular trabeculae from healthy rat hearts contracting at various stimulation frequencies under physiological conditions. Trabeculae were chosen for this study because of their linear cardiomyocyte arrangement and thin structure, which minimises the risk of hypoxia even during high metabolic demand (Choi et al., 2020; Han et al., 2011). We used a flow‐through calorimeter to measure force–length work‐loops and heat output in isolated ventricular trabeculae. Two contraction protocols (isometric length change and afterload work‐loop) were employed to partition various energy expenditure sources, including cross‐bridge activity and Ca^2+^ cycling‐related activation processes. Measurements were made over ranges of afterloads and muscle lengths to quantify peak energy efficiency. Our results provide insights into intrinsic cardiac muscle energetics as functions of heart rate.

## 2 METHODS

### 2.1 Muscle preparation

The experiments were conducted in accordance with protocols approved by the University of Auckland Animal Ethics Committee (AEC22653). Male Wistar rats (8–10 weeks old, 250–350 g) were purchased from the animal facility (Vernon Jansen Unit) at the University of Auckland. The rats were housed in a 12/12 light–dark cycle at room temperature (21–22°C) and fed standard rat chow and tap water ad libitum. Each rat was brought to our department (Auckland Bioengineering Institute within the University of Auckland) and placed in a climate‐control chamber with access to food and water for at least 1 h to minimise any stress arising from the transportation. Rats were anaesthetised with isoflurane (5% in O_2_) before they were injected with heparin (1000 IU kg^−1^). Following a cervical dislocation, the heart was excised and arrested with cold (4°C) Tyrode–BDM solution, which contained (in mmol L^−1^): 130 NaCl, 6 KCl, 1 MgCl_2_, 0.5 NaH_2_PO_4_, 0.3 CaCl_2_, 10 HEPES, 10 glucose and 20 2,3‐butanedione monoxime (BDM), pH adjusted to 7.4 using Tris at room temperature. The heart was immediately Langendorff‐perfused with the same Tyrode–BDM solution, continuously bubbling with 100% oxygen, at room temperature.

Under a dissecting microscope, intact trabeculae were dissected from the endocardial surface of the left ventricle. A geometrically suitable trabecula was then mounted in a work‐loop calorimeter (Mellor et al., 2021; Taberner et al., 2019) and held by two platinum hooks. One ‘upstream’ hook was connected to a linear motor for controlling muscle length, and another ‘downstream’ hook was connected to a custom‐built force transducer. In the measurement chamber of the calorimeter, the muscle was superfused along its length from upstream to downstream direction with the oxygenated Tyrode solution containing no BDM and a higher concentration of CaCl_2_ (1.5 mmol L^−1^). The pH of Tyrode superfusate was adjusted to 7.4 with Tris at 37°C. The flow rate of superfusate was electronically maintained at 0.55 µL s^−1^; this flow rate provided adequate oxygenation (Han et al., 2011) while maximising the thermal signal‐to‐noise ratio (Johnston et al., 2015; Taberner et al., 2015).

The rate of muscle heat output was measured and quantified from the difference in temperature between downstream and upstream thermopile arrays and the flow‐rate‐dependent temperature sensitivity (Taberner et al., 2018). The calorimeter was enclosed in an optically isolated and thermally insulated cabinet. The environmental temperature in the enclosure was gradually elevated within 1 h and was maintained at 37°C during experiments.

### 2.2 Experimental protocols

The trabecula was electrically stimulated at 2 Hz via two platinum electrodes located proximate to the calorimeter measurement chamber. Once a steady state of twitch force development was reached, the muscle was gradually stretched stepwise (100 µm increment) using the upstream linear motor. The optimal length (*L*_o_) was determined where the muscle developed the highest twitch force. The trabecula underwent two contraction‐mode protocols: afterloaded work‐loop contractions at *L*_o_ and isometric contractions at various muscle lengths. In each protocol, stimulus frequency was presented in a random order (2 Hz, 3.5 Hz and 5 Hz).

The trabecula was first required to perform afterloaded work‐loop contractions. This protocol aimed to evaluate force–length work output, change of enthalpy and mechanical efficiency over a range of afterloads. In this protocol, the trabecula was stimulated to contract isometrically at *L*_o_, where its length was servo‐maintained constant using the upstream length motor. The trabecula was next required to undergo force–length work‐loop contractions at an afterload before switching back to isometric contractions. The protocol was repeated for five decreasing afterloads ranging from ∼80% to ∼5% of steady‐state active twitch stress. At each afterload, the trabecula reached a steady state of shortening and heat output after approximately 3 min. The trabecula was returned to isometric contractions at *L*_o_ between each bout of work‐loops to allow comparison of the baselines of the force and the rate of heat production at isometric contractions. Force–length work‐loop contractions approximated the pressure–volume loops of the heart, thereby allowing measurements of extent of shortening, velocity of shortening and mechanical work output (Loiselle et al., 2014; Pham et al., 2017). The work‐loop protocol was repeated at the other two stimulus frequencies while electrical stimulation was halted between each frequency intervention, thereby providing baselines of force and heat.

Upon completion of the work‐loop protocol, the trabecula was then subjected to a varying‐length isometric protocol, where it underwent a series of isometric contractions at five different muscle lengths (*L*_o_, *L*_95_, *L*_90_, *L*_85_, *L*_80_, where *L*_80_ is at 80% of *L*_o_). This protocol aimed to assess twitch force kinetics and the heat–stress relation. Electrical stimulation was halted at each length‐step transition to provide baselines for zero force and heat. At each length‐step, stimulus frequency was changed. Measurements of force, length and heat rate were acquired using LabVIEW software (National Instruments, Austin, TX, USA) and analysed offline using a custom‐written MATLAB script (MathWorks, Natick, MA, USA).

At the end of the experiments, two sources of heat artefact were quantified. While the muscle was made quiescent by halting electrical stimulation, the heat artefact arising from the small cyclic movement (up to about 0.3 mm) of the upstream hook (required to change muscle length during work‐loop contractions by the upstream length‐motor) was quantified. This movement heat artefact was measured by oscillating the upstream hook at each stimulus frequency at the maximum extent of muscle shortening obtained during work‐loops. The second heat artefact, resulting from electrical stimulation at each frequency, was quantified in the absence of the muscle. The muscle was removed from the calorimeter, and stimulus heat artefact was measured at three stimulus frequencies. Subtracting both heat artefacts revealed the net rate of muscle heat output.

### 2.3 Trabecula geometry

In total, seven LV trabeculae were isolated from four rats and studied in this experiment. Since each studied trabecula resembled an ellipse in cross‐section, cross‐sectional areas were calculated from the two orthogonal measurements of diameters. The optimal length of the trabecula (*L*_o_) was measured in the calorimeter, and muscle diameters in two orthogonal views were measured via a 45° mirror, using a microscope graticule. The mean ± SD muscle cross‐sectional area was 0.061 ± 0.028 mm^2^ and the mean ± SD muscle length was 2.89 ± 0.30 mm.

### 2.4 Normalisations and definitions

Force was converted to stress (kPa) by normalising to muscle cross‐sectional area. Twitch duration was quantified at 5% of the peak twitch stress. The maximum rates of rise and fall of twitch stress were computed from the ascending and descending limbs of the twitch, respectively. Heat per twitch, or active heat (kJ m^−3^), was calculated by dividing the steady‐state heat rate by the stimulus frequency and normalising it to muscle volume. Active heat comprises the thermal output from the splitting of ATP required for two cellular activities: Ca^2+^ cycling (activation heat) and cross‐bridge cycling (cross‐bridge heat). Each heat–stress relation was fitted using linear regression for each frequency, where isometric heat intercept quantifies activation heat, and the reciprocal of the slope of the relation provides a measure of the contractile economy. Afterload was the stress at which the muscle was required to transition from the isometric phase to the isotonic shortening phase of the work‐loop. Relative afterload was the ratio of the afterload to the peak isometric total (active plus passive) stress. Relative extent of shortening (%) was determined by the distance that the muscle shortened during the isotonic phase at each afterloaded work‐loop and was expressed relative to *L*_o_. Relative velocity of shortening (s^−1^) was calculated as the maximal slope of the relative length–time trace during the isotonic phase of each afterloaded work‐loop. Mechanical work output per twitch (kJ m^−3^) was calculated as the area within the work‐loop by integrating stress as a function of relative muscle length over the period of the twitch. Change of enthalpy (kJ m^−3^) was calculated as the sum of work and heat. Mechanical efficiency (%) was defined as the ratio of work to change of enthalpy.

### 2.5 Statistical analyses

Data from individual trabeculae were fitted using polynomial regressions (up to third order). Peak values of parameters of interest for each trabecula were computed from each regression line. Regression lines were averaged across all muscles using the random coefficient model within PROC MIXED of the SAS software package (SAS Institute Inc., Cary, NC, USA). Significant differences between peak values were tested for the effect of frequency using a one‐way repeated‐measures ANOVA in GraphPad Prism (Version 9, GraphPad Software, Boston, MA, USA), with *post hoc* Tukey analysis. The statistical significance of a difference was declared when *P* < 0.05. Peak values of parameters were plotted in bars and expressed as means ± standard errors unless specified otherwise.

## 3 RESULTS

### 3.1 Typical experimental records

**FIGURE 1:**
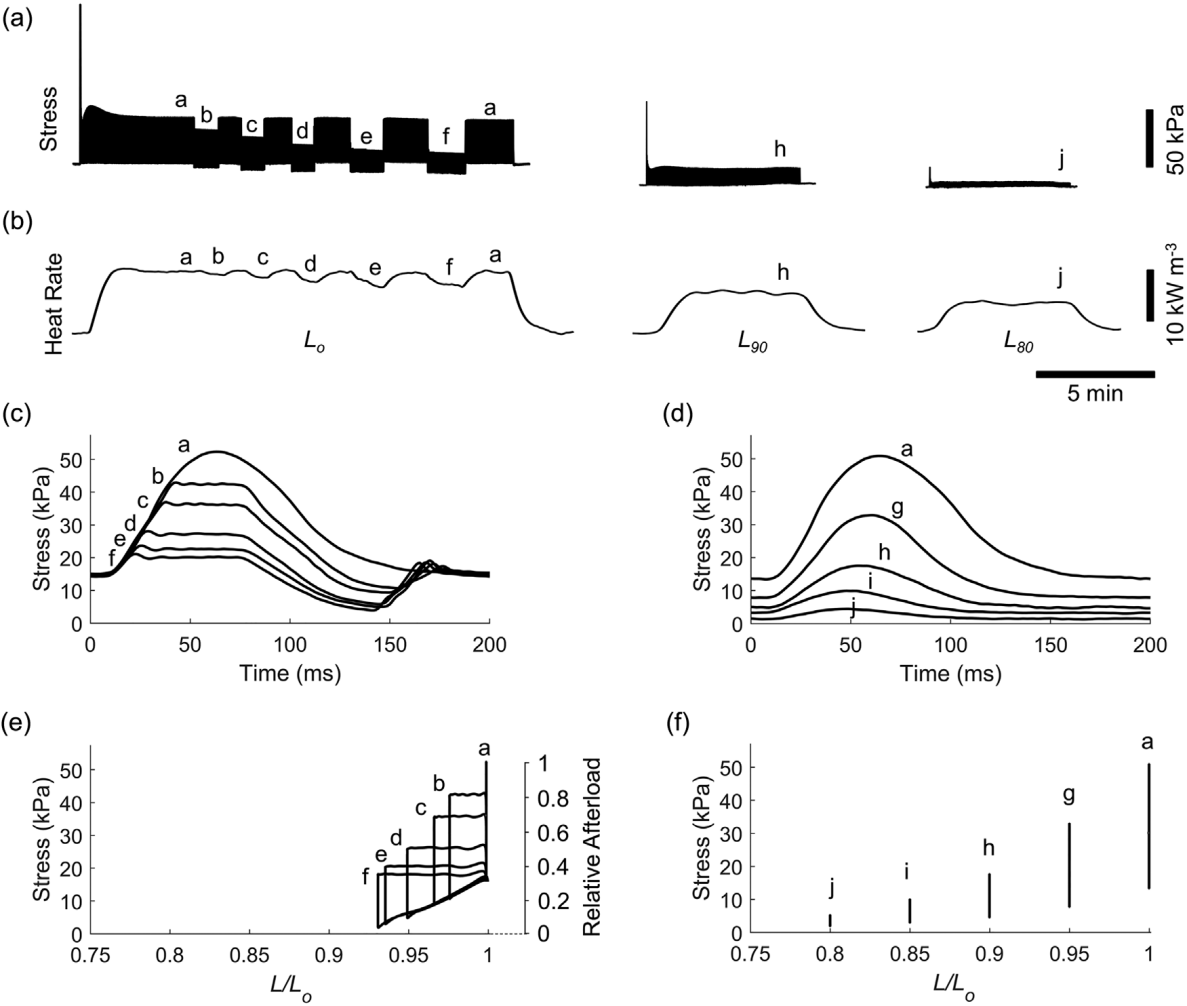
Experimental records and post‐experimental analysis. Twitch stress (a) and rate of heat production (b) of a representative trabecula contracting at 5 Hz and undergoing progressively decreasing afterloaded work‐loop contractions (labelled *b–f*), bounded by isometric contractions at *L*_o_ (labelled *a*). The first twitch illustrates the rested‐state contraction immediately following the commencement of electrical stimulation. Steady‐state twitch stress profiles at various afterloads under work‐loop contractions (c) and at various muscle lengths under isometric contractions (d). (e) A parametric plot of stress and length (relative to *L*_o_) of the work‐loop protocol to illustrate after‐loaded work‐loops (labelled *b–f*) and the isometric contraction (labelled *a*). (f) A parametric plot of stress and length (relative to *L*_o_) of the isometric length‐change protocol.

Figure 1 presents simultaneous measurements of developed twitch stress, rate of heat output, and length change of a representative trabecula contracting at 5 Hz. In the work‐loop protocol, as stress (afterload) decreased progressively from *b* to *f*, there was a corresponding reduction in heat output (Figure 1a, b). The steady‐state work‐loop twitches at all afterloads are superimposed and presented in Figure 1c to illustrate the afterload decrease from *b* to *f*, relative to the isometric stress labelled *a*. A parametric plot of stress and length data for each afterloaded work‐loop contraction at a steady state is presented in Figure 1e to illustrate work‐loops. No work output was performed under isometric condition *a*, and heat output was the greatest under isometric condition compared with those under work‐loops from *b* to *f*.

In the isometric length‐change protocol, reducing muscle length (labelled *a–j*) led to a decrease in both steady‐state active stress (Figure 1a) and active heat output (Figure 1b). Figure 1d shows the superimposed steady‐state isometric twitches across the five muscle lengths, ranging from optimal length (*L*_o_; labelled *a*) to 80% of the optimal length (*L*_80_; labelled *j*), as depicted in Figure 1f.

### 3.2 Work‐loop contractions

**FIGURE 2:**
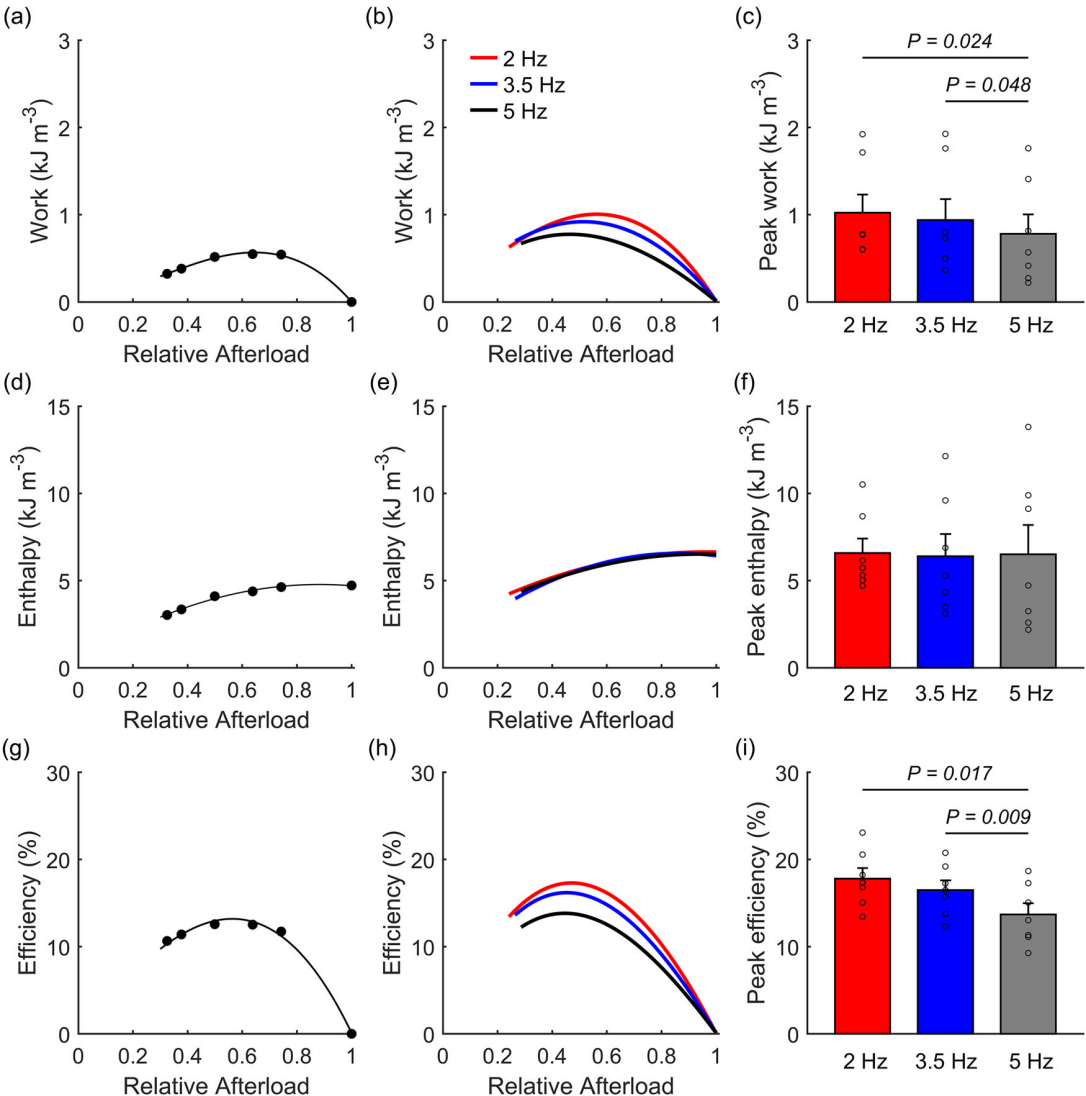
Energetics of work‐loop contractions at steady state. Work output, change of enthalpy and mechanical efficiency plotted as functions of relative afterload of a single trabecula contracting at 5 Hz (a, d, g) or the mean relations of all trabeculae (b, e, h) respectively). Data were fitted using second‐order polynomials for change of enthalpy and third‐order polynomials for the work and efficiency as functions of relative afterloads. *P < 0.05. Bar plots present means ± SEM, *n* = 7.

Figure 2a, d and g display representative data from the work‐loop protocol showing steady‐state twitch work, change of enthalpy and mechanical efficiency as functions of relative afterloads, obtained from a representative trabecula contracting at 5 Hz (filled circles) under various afterloads. Peak values of work, change of enthalpy and mechanical efficiency were computed from the curve fitting for this representative trabecula. The average curves of all trabeculae are depicted in Figures 2b, e and h. Our data show no statistically significant difference in the relative afterloads at which peak work or efficiency occurs among frequencies.

The mean peak values for work output per twitch (Figure 2c) and mechanical efficiency (Figure 2i) at 5 Hz were significantly lower than those at 2 and 3.5 Hz, with no differences detected between 2 and 3.5 Hz. However, no differences were observed in the mean peak change of enthalpy across the different frequencies (Figure 2f).

**FIGURE 3:**
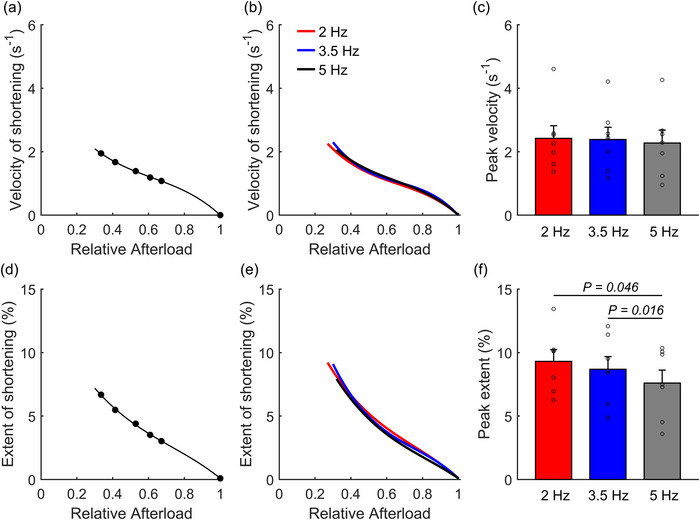
Shortening‐related parameters arising from the work‐loop protocol. (a, d) The extent of shortening and velocity of shortening plotted as functions of relative afterload from a representative trabecula contracting at 5 Hz, from which peak values were computed. Data were fitted using third‐order polynomials. (b, e) The relations averaged across all trabeculae. (c, f) Peak values of velocity of shortening (c) and extent of shortening (f). Peak values were predicted from the fitted curves at the afterload where active stress is zero, i.e., in the vicinity of passive stress. *P < 0.05. Bar plots present means ± SEM, n = 7.

Velocity of shortening and extent of shortening were plotted against relative afterload and fitted with third‐order polynomial regression (Figure 3). Peak values for both metrics were identified at the lowest afterload, where passive stress occurred, and active stress was zero. There were no significant differences in peak velocity of shortening between stimulus frequencies (Figure 3c). However, the mean peak extent of shortening at 5 Hz was significantly lower than those at 2 and 3.5 Hz, with no difference between 2 and 3.5 Hz (Figure 3f).

### 3.3 Isometric contractions

**FIGURE 4:**
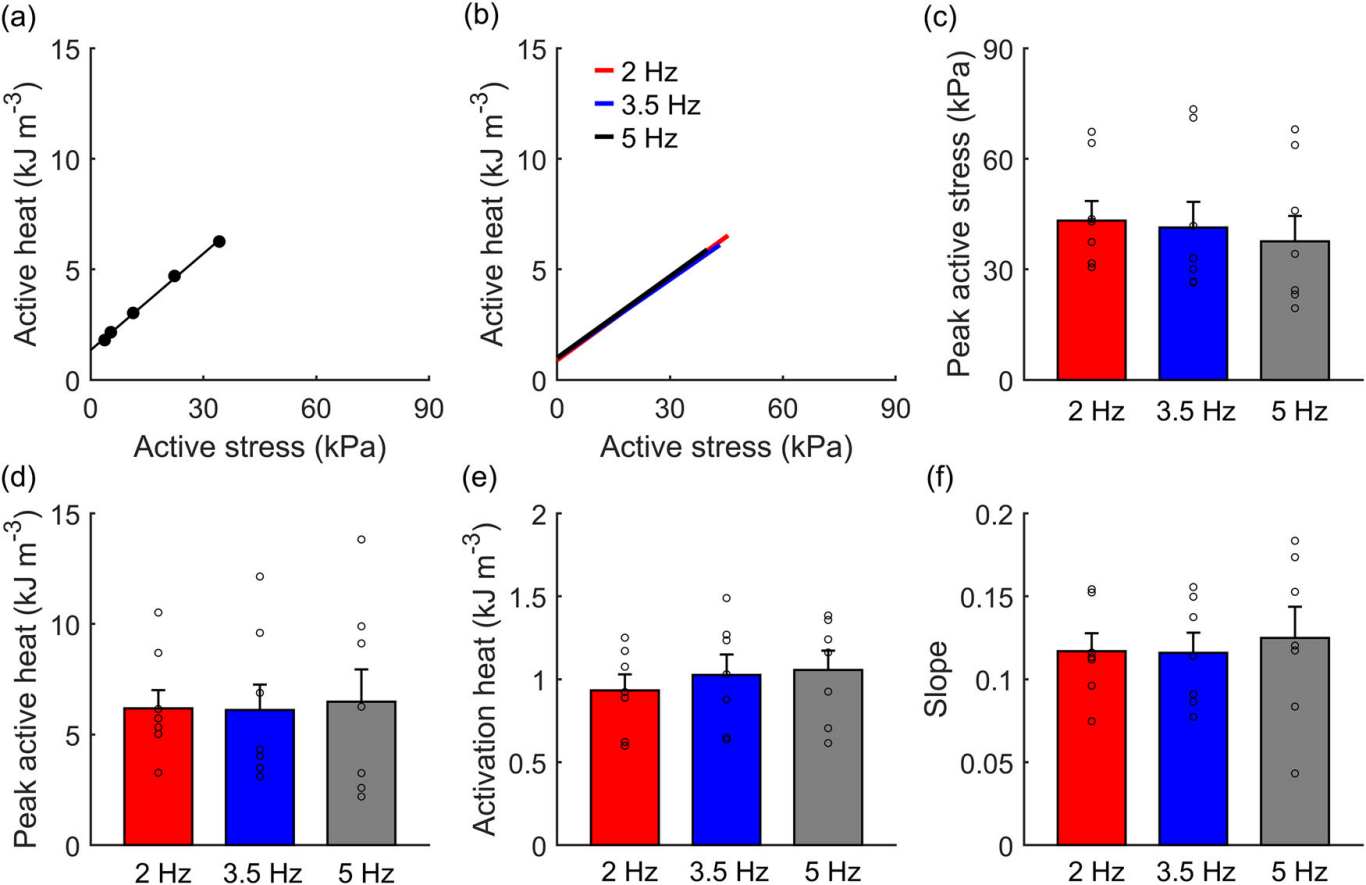
Energetics of isometric contractions at steady state. (a) Linear regression fitted to the heat–stress data from the isometric length‐change protocol at 5 Hz (filled circles) for a representative trabecula. (b) The heat–stress relations averaged across all trabeculae. (c, d) Peak values of active stress (obtained at *L*_o_) (c) and peak values of active heat (obtained at *L*_o_) (d). (e, f) The heat‐intercept (‘activation heat’) (e) and the active heat–stress relation slope (f) averaged across all trabeculae. All parameters were not significantly different among stimulus frequencies. Bar plots present means ± SEM, *n* = 7.

Figure 4a displays representative data from a single representative trabecula contracting at 5 Hz. Steady‐state twitch heat was plotted as a function of active stress obtained from the isometric length‐change protocol. The data were fitted using linear regression. Figure 4b shows the mean regression lines across all trabeculae and at the three stimulus frequencies. No significant differences were detected among stimulus frequencies in peak active stress and peak active heat, which were obtained at *L*_o_ (Figure 4c and d, respectively), as well as both diastolic and systolic stress (data not shown). The heat‐intercept of the active heat–stress relation provides an estimate of activation heat (Pham et al., 2017); no differences in the mean activation heat were observed among stimulus frequencies (Figure 4e). Likewise, no differences were detected among stimulus frequencies in the slope of the active heat–stress relation (Figure 4f).

**FIGURE 5:**
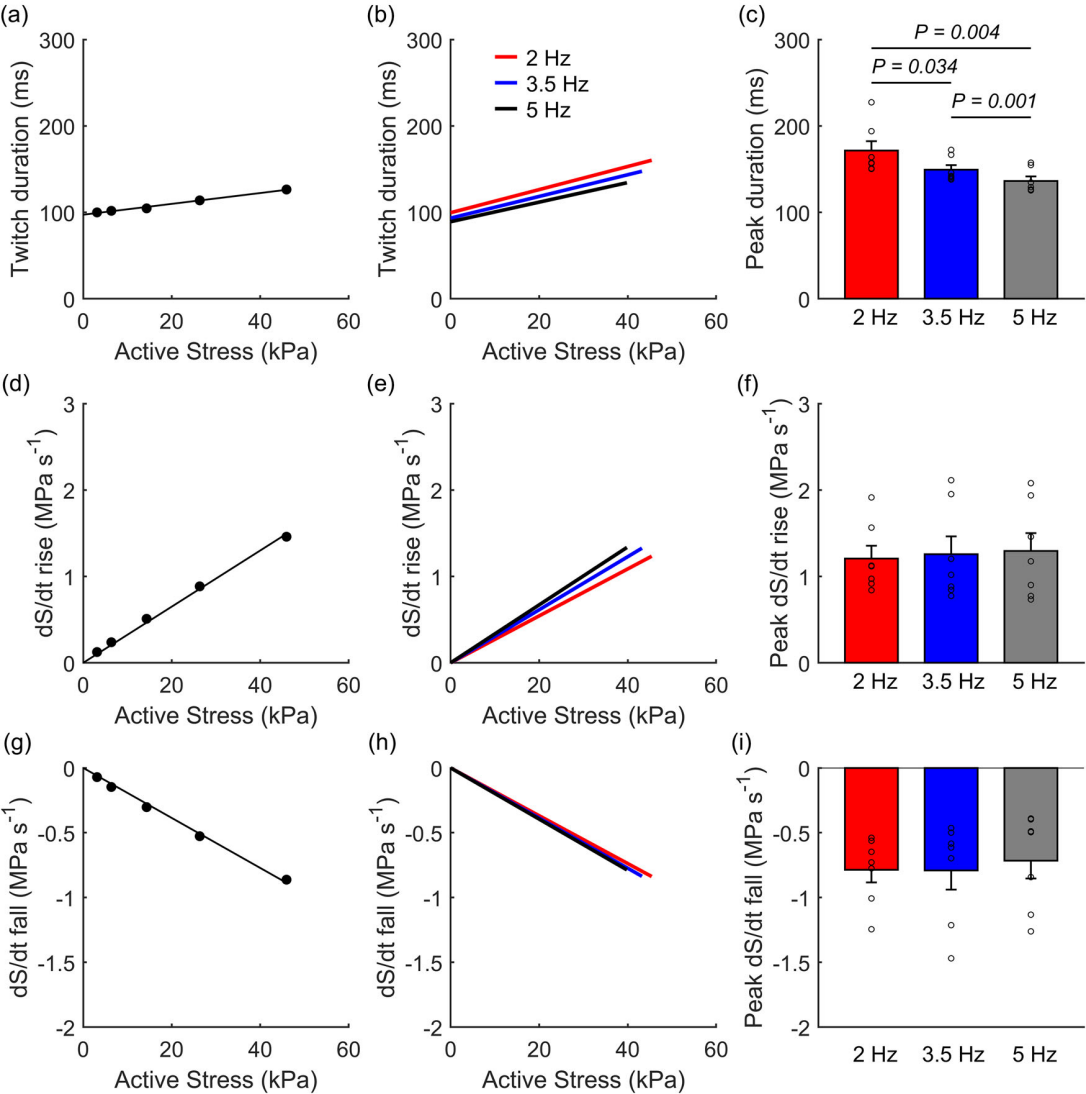
Kinetics of isometric twitches at steady state. (a, d, g) Linear regression of steady‐state isometric twitch duration (a) and maximum rates (d*S*/d*t*) of rise (d) and fall (g) of twitches as functions of active stress of a representative trabecula contracting at 5 Hz. (b, e, h) The corresponding mean relations across all trabeculae. (c, f, i) Mean peak values. *P < 0.05. Bar plots present means ± SEM, *n* = 7.

The mean peak twitch duration in steady‐state isometric twitches was the shortest at 5 Hz, followed by 3.5 and 2 Hz (Figure 5). No differences were found in the peak rates of rise and fall (d*S*/d*t* rise and fall) between the frequency groups.

### 3.4 Correlations among energetics parameters

**FIGURE 6:**
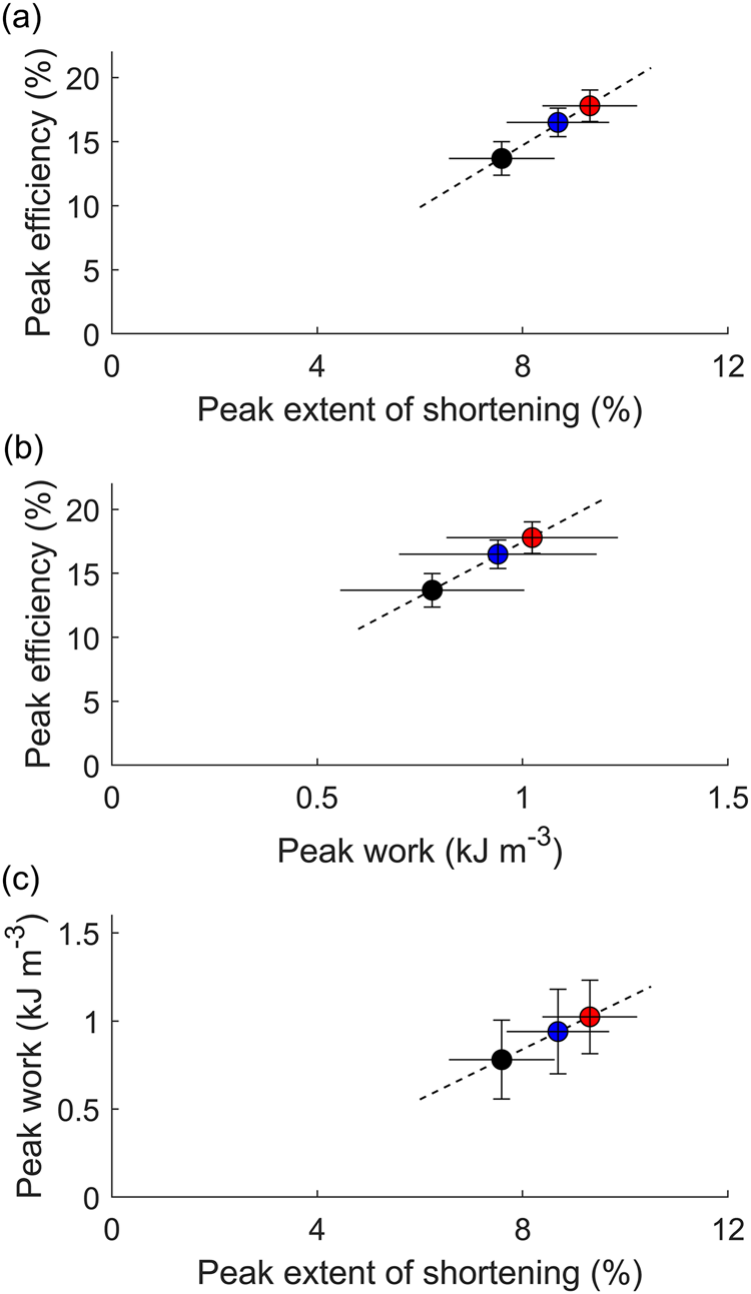
Correlations among mean peak values of mechano‐energetic parameters. The dashed line in each panel is fitted to the three data points denoting 2 Hz (red circle), 3.5 Hz (blue circle) and 5 Hz (black circle). Symbols are means ± SEM, *n* = 7.

Peak mechanical efficiency was lower at 5 Hz compared to those at 2 Hz and 3.5 Hz. Figure 6 shows the correlations among mean peak mechano‐energetic parameters as functions of frequency. Our data show that the mean peak mechanical efficiency was positively correlated with either the mean peak extent of shortening or work output. The mean peak work was positively correlated with the mean peak extent of shortening.

## 4 DISCUSSION

This study is the first to examine the effect of contraction frequency on mechano‐energetics of left ventricular trabeculae isolated from healthy rats using physiologically relevant protocols and experimental conditions. The novel approach includes subjecting the trabeculae to a full range of afterloads, enabling us to determine peak work output, mechanical efficiency, shortening velocity and extent at three contraction frequencies, i.e. 2 Hz, 3.5 Hz and 5 Hz. Our key findings reveal that lower contraction frequencies (2 Hz and 3.5 Hz) confer an energetic advantage to trabeculae for achieving higher peak work per twitch with no increase in change of enthalpy compared to 5 Hz. This increased work output results from a greater shortening extent without a concomitant change in shortening velocity or stress production. Additionally, the isometric contraction protocol showed no difference in activation heat between frequencies nor any change in isometric stress, heat or kinetics as a function of active stress. These results underscore the beneficial effect of lower contraction frequencies on cardiac muscle energy efficiency.

### 4.1 Experimental design

We isolated cardiac trabeculae from healthy rat hearts to test whether varying heart rates directly impact energy efficiency. Exposing isolated trabeculae to precisely controlled afterloads eliminates in vivo confounding factors such as increased peripheral resistance (Julius, 1988) and activated sympathetic activity (Grassi et al., 1998). These have been observed in disease settings and considered as compensatory mechanisms to preserve cardiovascular homeostasis (Triposkiadis et al., 2009). These factors can obscure the direct effects of stimulation frequency on cardiac performance. By avoiding these confounders, we sought a more direct evaluation of how contraction frequencies influence the intrinsic energetic performance of cardiac muscle.

Isolated cardiac preparations are known to gradually decline in force development over time; this phenomenon is particularly pronounced in experiments at body temperature (Pham et al., 2019). This force ‘rundown’ is likely due to progressive time‐dependent decreases in sarco/endoplasmic reticulum Ca^2+^‐ATPase (SERCA) activity (Taylor et al., 2004) and sarcoplasmic reticular Ca^2^⁺ load (Milani‐Nejad et al., 2014), both of which are critical for contractile force development (Bers, 2002; Maier et al., 2000). The force decline presents a significant challenge in experimental design, as it can confound the interpretation of results. We implemented a randomised order of stimulation frequencies across the contraction protocols to address this issue. Our approach ensured that any time‐dependent force decline was evenly distributed across all frequencies and allowed for accurate assessments of the intrinsic relationship between contraction frequency and cardiac mechano‐energetic performance.

### 4.2 Isometric stress

The stress–frequency relationship in rat myocardium was reported to be positive (Layland & Kentish, 1999; Shen et al., 2013), biphasic (Forester & Mainwood, 1974; Janssen et al., 2003; Pham et al., 2019; Tang et al., 1996; Taylor et al., 2004), negative (Han et al., 2010; Johnston et al., 2016; Taylor et al., 2004) or flat (Layland & Kentish, 2000; Schouten & ter Keurs, 1986). These inconsistencies likely stem from differences in experimental conditions, particularly the interaction between extracellular calcium concentration ([Ca^2+^]_o_) and temperature. For instance, a study conducted at 26°C using rat left ventricular papillary muscles observed a negative stress–frequency relationship at high [Ca^2+^]_o_ (2.5 mmol L^−1^) but a biphasic relationship at low [Ca^2+^]_o_ (1 mmol L^−1^) (Forester & Mainwood, 1974). Similar results were found in rat right ventricular trabeculae, showing a more negative relation at high [Ca^2+^]_o_ (2 mmol L^−1^) compared to that of 1 mmol L^−1^ at 22°C (Han et al., 2010). Additionally, the stress–frequency relationship was shown to become more negative at 27°C compared to 37°C, even at physiological [Ca^2+^]_o_ level (1.5 mmol L^−1^) (Johnston et al., 2016). Another study indicated a shift from a negative to a biphasic profile after 3 h of Ca^2+^ dye Fura‐2 loading in rat papillary muscles (Taylor et al., 2004). The observed negative or flat stress–frequency relationships in rat myocardium may be explained by an increase in the refractoriness of sarcoplasmic reticulum Ca^2^⁺ release and a decrease in the magnitude of the trigger for Ca^2^⁺ release, assuming minimal change in SR Ca^2^⁺ content with increasing frequency (Bouchard & Bose, 1989; Maier et al., 2000). These variations feature the complex and condition‐dependent nature of the stress–frequency relationship, highlighting the need for carefully controlled experimental conditions when assessing cardiac function.

In this study, we conducted experiments at physiological temperature and [Ca^2+^]_o_ using isolated rat left ventricular trabeculae. Our findings of a flat stress–frequency relationship over the 2 and 5 Hz range align with previous results under similar experimental conditions (Pham et al., 2019) and at sub‐physiological temperatures (Layland & Kentish, 2000; Schouten & ter Keurs, 1986). In contrast to rat myocardium, a positive force–frequency relation in cardiac preparations has been widely reported in other species, including rabbit (Maier et al., 2000), dog (Rouleau et al., 1986; Taylor et al., 2004) and human (Pieske et al., 1999; Taylor et al., 2004), commonly attributed to the increased SR Ca^2+^ load resulting from differences in speed of uptake between SERCA and the Na⁺/Ca^2^⁺ exchanger (NCX) (Bers, 2000; Maier et al., 2000). The lack of a positive force–frequency relationship often seen in rodents likely reflects the dominance of SERCA even at low frequencies.

### 4.3 Active heat

Energy expenditure of cardiac muscle in isometric contraction appears entirely as heat production due to the absence of macroscopic work. Isometric heat production comprises two main components: activation heat and cross‐bridge heat. Activation heat represents the energy required for cellular Ca^2^⁺ cycling, primarily driven by SERCA with additional contributions from the sarcolemmal NCX and Na⁺‐K⁺ ATPase. Activation heat can be precisely quantified as the heat‐intercept of the isometric heat–stress relation (Pham et al., 2017). Our findings reveal that activation heat is independent of stimulation frequency, consistent with previous studies (Han et al., 2010; Johnston et al., 2016). This result is also consistent with previous studies that reported similar magnitudes of Ca^2^⁺ transients across different stimulation frequencies in rat ventricular trabeculae (Litwin & JP, 1999; Maier et al., 1998), although these studies were conducted at room temperature.

Frequency‐dependent acceleration of twitch relaxation is an intrinsic mechanism of the heart. Various Ca^2+^‐dependent kinases or phosphatases such as myosin light chain kinase, calcineurin and Ca^2^⁺–calmodulin kinase (CaMKII) are activated in response to increased stimulation rates. CaMKII plays a key role in this process by enhancing sarcoplasmic reticular calcium uptake, enabling faster twitch relaxation as heart rate increases (Bassani et al., 1995). This aligns with earlier findings showing faster relaxation of Ca^2^⁺ transient twitch at higher stimulation frequencies (Maier et al., 1998). However, similar magnitudes of Ca^2^⁺ transients across different frequencies (Litwin & JP, 1999; Maier et al., 1998) may suggest comparable energy expenditure for calcium reuptake to diastolic levels. The amount of Ca^2^⁺ released from the sarcoplasmic reticulum dictates the extent of its sequestration by SERCA, and our thermal data, where we found no frequency effects on activation heat (Figure 4), align with the frequency‐independent Ca^2+^ transient amplitude.

Cross‐bridge heat originates from the ATP hydrolysis by actin‐activated myosin ATPase during the cross‐bridge cycling required for force generation. Cross‐bridge heat can be determined by subtracting activation heat from the total active heat. Our data show no difference in total active heat between frequencies, indicating that the energy required for activation processes and cross‐bridge detachment and reattachment does not vary with stimulus frequency. Our heat findings align with a previous report on rat left‐ventricular and right‐ventricular trabeculae under comparable experimental conditions (Pham et al., 2019).

### 4.4 Work output per twitch

The contraction protocol in this study enabled us to determine the peak force–length work per twitch produced by trabeculae across a range of afterloads. Peak work occurred at a relative afterload of 0.5–0.6 (Figure 2). At this mid‐range afterload, peak work output per twitch at 5 Hz was significantly lower than at 2 and 3.5 Hz, with no difference between 2 and 3.5 Hz. These results align with studies showing higher work output at lower stimulation frequencies in rat trabeculae (Han et al., 2013) and papillary muscles (Mellors et al., 2001). However, those studies were conducted at room temperature and with lower frequency ranges. In this study, we did not explore frequencies higher than 5 Hz. Our protocol determined the optimal muscle length (*L*_o_) by progressively stretching the muscle until active force ceases to increase, ensuring a consistent reference muscle length for maximum force production across all samples. By conducting experiments at *L*_o_, we maximised muscle contractile force and heat production, aligning with previous findings from our group showing that trabeculae operate at peak mechanical efficiency at this length (Han et al., 2022). However, this approach restricts us from conducting experiments at frequencies above 5 Hz due to the lack of a diastolic phase, preventing the completion of work‐loop contractions. Nevertheless, whereas the in vivo heart rate of the rat is 5–6 Hz (300–360 bpm), the ex vivo and in vitro rat heart beats intrinsically at around 2.5–3.5 Hz (150–210 bpm), as reported in the literature (Stanford et al., 1990) and in our own studies (Han et al., 2014; Han et al., 2015; Han et al., 2018). Thus, our findings from the present study contribute to the clinical benefit of reducing heart rate, which is typically restoring a high resting heart rate to a healthy range, at least for ex vivo and in vitro hearts.

Previous studies using isotonic sinusoidal contraction protocols have explored a full range of stimulation frequencies on work output in rat cardiac preparations. Investigations on rat papillary muscles found that the highest work occurred at 2–2.5 Hz within a frequency range between 1 Hz and 4 Hz at room temperature (Baxi et al., 2000) and at 3–4 Hz within a range between 1 Hz and 7 Hz at 37°C (Layland et al., 1995). Similar findings were observed in rat trabeculae, with maximal work at 2 Hz over a 0.5–6 Hz range at room temperature (Layland & Kentish, 2002). These work–frequency profiles are consistent with our results, which show no significant difference in work output between 2 Hz and 3.5 Hz but a decrease at 5 Hz. Previous studies reported that at increasing frequencies, diastolic Ca^2+^ levels in rat cardiac muscles significantly increase while myofilament Ca^2+^ sensitivity decreases (Varian & Janssen, 2007). In addition, faster intracellular Ca^2+^ decline with increasing stimulation rates would reduce the time available for cross‐bridge activation and further increase the speed of relaxation (Janssen et al., 2002; Lamberts et al., 2007). However, our data show no statistical evidence that there is an increased diastolic stress due to incomplete twitch relaxation at the higher frequencies. The lower peak work observed at 5 Hz, despite no difference in stress production across frequencies, can be attributed to the lower extent of muscle shortening due to the shorter time duration per twitch at this higher frequency (Figure 5).

The muscle has more time per contraction cycle at lower frequencies, allowing for more shortening, as the shortening velocity remains consistent across all frequencies (Figure 3). If we consider muscle shortening as a proxy for stroke volume (Glower et al., 1985), we expect a lower stroke volume at 5 Hz compared to 2 Hz and 3.5 Hz. This speculation aligns with a previous study that reported a negative correlation between stroke volume and heart rate in the ex vivo working rat heart under increasing temperature (Power et al., 2014). A clinical study reported that lower heart rates, induced with zatebradine, increased stroke volume index and work output in patients with left ventricular dysfunction (Shinke et al., 1999).

### 4.5 Mechanical efficiency

Mechanical efficiency, defined as the ratio of work output to enthalpy expenditure, is a key measure of cardiac performance. Inconsistent findings on cardiac energy efficiency have been reported in the literature, with some studies reporting lower (Baxi et al., 2000), no difference or higher efficiency (Baxi et al., 2000; Mellors et al., 2001) at higher frequencies. The inconsistency may stem from the lack of physiological conditions, such as temperature, contraction frequency and realistic shortening protocols, that mimic in vivo ejection. Those studies often used sub‐physiological temperatures (Baxi et al., 2000; Han et al., 2013, 2010; Mellors et al., 2001), frequencies (Han et al., 2013; Mellors et al., 2001), sinusoidal contraction (Baxi et al., 2000; Mellors et al., 2001) or isometric contraction modes (Han et al., 2010; Pham et al., 2019). Our experimental design allowed us to assess the intrinsic behaviour of isolated cardiac preparations under physiological experimental conditions. Our findings show that peak mechanical efficiency was lower at 5 Hz compared to 2 Hz and 3.5 Hz, reflecting lower work output with no change in total enthalpy, consistent with previous reports (Baxi et al., 2000; Han et al., 2013). The peak mechanical efficiency values in this study are in the range of 10–20% and in line with earlier reports on sinusoidally contracting isolated rat papillary muscles (Barclay et al., 2003; Baxi et al., 2000; Mellors et al., 2001) and work‐loop contracting trabeculae (Mellor et al., 2021; Pham et al., 2017, 2018, 2022).

We assessed the correlations among energetic parameters in Figure 6. We show linear correlations between mean peak mechanical efficiency at various frequencies and their corresponding peak work or extent of shortening, as well as between peak work output and peak extent of shortening. Our findings support the notion that heart rate reduction has direct benefits for cardiac muscle energetics, at least in healthy myocardium, which is also observed in left ventricular dysfunction patients (Shinke et al., 1999). Our data do not show a distinct ‘threshold’ between 5 Hz and the lower frequencies (2 Hz and 3.5 Hz). Many of our data plots show a progressive change from 2 Hz to 3.5 Hz and to 5 Hz. Figure 6 further shows linear correlations among mechano‐energetic variables when all these three frequencies are assessed collectively. Given that these data in Figure 6 fit onto linear lines, we thus could not offer a mechanism for ‘transition that occurs’ for frequencies lower than 5 Hz.

### 4.6 Summary

Our findings demonstrate that increased work output per twitch, unchanged activation and cross‐bridge heat, and greater shortening extent are key factors leading to the higher mechanical efficiency observed at lower stimulation frequencies in contracting left‐ventricular trabeculae.

## AUTHOR CONTRIBUTIONS

All authors designed the study. T.P. performed the experiments and was responsible for the acquisition and statistical analysis of data, preparing figures and drafting the manuscript. A.T. was responsible for the maintenance of the calorimeter. A.T. and J.‐C.H. contributed to the interpretation and statistical analysis of data. All authors have read and approved the final version of this manuscript and agree to be accountable for all aspects of the work in ensuring that questions related to the accuracy or integrity of any part of the work are appropriately investigated and resolved. All persons designated as authors qualify for authorship, and all those who qualify for authorship are listed.

## CONFLICT OF INTEREST

None declared.

## Data Availability

The data that support the findings of this study are available from the corresponding author upon reasonable request.

## References

[eph13746-bib-0001] Asanoi, H. , Ishizaka, S. , Kameyama, T. , & Sasayama, S. (1994). Neural modulation of ventriculoarterial coupling in conscious dogs. The American Journal of Physiology, 266(2 Pt 2), H741–H748. 8141375 10.1152/ajpheart.1994.266.2.H741

[eph13746-bib-0002] Barclay, C. J. , Widen, C. , & Mellors, L. J. (2003). Initial mechanical efficiency of isolated cardiac muscle. Journal of Experimental Biology, 206(Pt 16), 2725–2732. 12847117 10.1242/jeb.00480

[eph13746-bib-0003] Bassani, R. A. , Mattiazzi, A. , & Bers, D. M. (1995). CaMKII is responsible for activity‐dependent acceleration of relaxation in rat ventricular myocytes. The American Journal of Physiology, 268(2 Pt 2), H703–H712. 7864197 10.1152/ajpheart.1995.268.2.H703

[eph13746-bib-0004] Baxi, J. , Barclay, C. J. , & Gibbs, C. L. (2000). Energetics of rat papillary muscle during contractions with sinusoidal length changes. American Journal of Physiology‐Heart and Circulatory Physiology, 278(5), H1545–H1554. 10775132 10.1152/ajpheart.2000.278.5.H1545

[eph13746-bib-0005] Bers, D. M. (2000). Calcium fluxes involved in control of cardiac myocyte contraction. Circulation Research, 87(4), 275–281. 10948060 10.1161/01.res.87.4.275

[eph13746-bib-0006] Bers, D. M (2002). Cardiac excitation–contraction coupling. Nature, 415(6868), 198–205. 11805843 10.1038/415198a

[eph13746-bib-0007] Bouchard, R. A. , & Bose, D. (1989). Analysis of the interval force relationship in rat and canine ventricular myocardium. American Journal of Physiology, 257(6 Pt 2), H2036–H2047. 2603987 10.1152/ajpheart.1989.257.6.H2036

[eph13746-bib-0008] Ceconi, C. , Cargnoni, A. , Francolini, G. , Parinello, G. , & Ferrari, R. (2009). Heart rate reduction with ivabradine improves energy metabolism and mechanical function of isolated ischaemic rabbit heart. Cardiovascular Research, 84(1), 72–82. 19477966 10.1093/cvr/cvp158

[eph13746-bib-0009] Ceconi, C. , Comini, L. , Suffredini, S. , Stillitano, F. , Bouly, M. , Cerbai, E. , Mugelli, A. , & Ferrari, R. (2011). Heart rate reduction with ivabradine prevents the global phenotype of left ventricular remodeling. American Journal of Physiology‐Heart and Circulatory Physiology, 300(1), H366–H373. 20952661 10.1152/ajpheart.01117.2009

[eph13746-bib-0010] Choi, D. H. , Pham, T. , Loiselle, D. S. , Taberner, A. J. , Han, J. C. , & Tran, K. (2020). The inverse relationship between cardiac muscle stress and cross‐sectional area is preserved in Ba^2+^ contracture and in chemically‐permeabilised Ca^2+^ contracture. Experimental Mechanics, 61, 107–117.

[eph13746-bib-0011] Colin, P. , Ghaleh, B. , Monnet, X. , Hittinger, L. , & Berdeaux, A. (2004). Effect of graded heart rate reduction with ivabradine on myocardial oxygen consumption and diastolic time in exercising dogs. The Journal of Pharmacology and Experimental Therapeutics, 308(1), 236–240. 14569053 10.1124/jpet.103.059717

[eph13746-bib-0012] DiFrancesco, D. , & Camm, J. A. (2004). Heart rate lowering by specific and selective I(f) current inhibition with ivabradine: A new therapeutic perspective in cardiovascular disease. Drugs, 64(16), 1757–1765. 15301560 10.2165/00003495-200464160-00003

[eph13746-bib-0013] Dobre, D. , Borer, J. S. , Fox, K. , Swedberg, K. , Adams, K. F. , Cleland, J. G. , Cohen‐Solal, A. , Gheorghiade, M. , Gueyffier, F. , O'Connor, C. M. , Fiuzat, M. , Patak, A. , Piña, I. L. , Rosano, G. , Sabbah, H. N. , Tavazzi, L. , & Zannad, F. (2014). Heart rate: A prognostic factor and therapeutic target in chronic heart failure. The distinct roles of drugs with heart rate‐lowering properties. European Journal of Heart Failure, 16(1), 76–85. 23928650 10.1093/eurjhf/hft129

[eph13746-bib-0014] Fagard, R. (2003). Athlete's heart. Heart, 89, 1455–1461. 14617564 10.1136/heart.89.12.1455 PMC1767992

[eph13746-bib-0015] Ferrari, R. , & Fox, K. (2016). Heart rate reduction in coronary artery disease and heart failure. Nature Reviews Cardiology, 13(8), 493–501. 27226153 10.1038/nrcardio.2016.84

[eph13746-bib-0016] Forester, G. V. , & Mainwood, G. W. (1974). Interval dependent inotropic effects in the rat myocardium and the effect of calcium. Pflugers Archiv: European Journal of Physiology, 352, 189–196. 4475405 10.1007/BF00590484

[eph13746-bib-0017] Fragasso, G. , De Cobelli, F. , Spoladore, R. , Esposito, A. , Salerno, A. , Calori, G. , Montanaro, C. , Maranta, F. , Lattuada, G. , Margonato, A. , Del Maschio, A. , & Perseghin, G. (2011). Resting cardiac energy metabolism is inversely associated with heart rate in healthy young adult men. American Heart Journal, 162(1), 136–141. 21742100 10.1016/j.ahj.2011.04.012

[eph13746-bib-0018] Glower, D. D. , Spratt, J. A. , Snow, N. D. , Kabas, J. S. , Davis, J. W. , Olsen, C. O. , Tyson, G. S. , Sabiston, D. C., Jr., & Rankin, J. S. (1985). Linearity of the Frank‐Starling relationship in the intact heart: The concept of preload recruitable stroke work. Circulation, 71(5), 994–1009. 3986986 10.1161/01.cir.71.5.994

[eph13746-bib-0019] Grassi, G. , Vailati, S. , Bertinieri, G. , Seravalle, G. , Stella, M. L. , Dell'Oro, R. , & Mancia, G. (1998). Heart rate as marker of sympathetic activity. Journal of Hypertension, 16(11), 1635–1639. 9856364 10.1097/00004872-199816110-00010

[eph13746-bib-0020] Han, J. C. , Barrett, C. J. , Taberner, A. J. , & Loiselle, D. S. (2015). Does reduced myocardial efficiency in systemic hypertensive‐hypertrophy correlate with increased left‐ventricular wall thickness? Hypertension Research, 38(8), 530–538. 25787044 10.1038/hr.2015.37

[eph13746-bib-0021] Han, J. C. , Goo, S. , Barrett, C. J. , Mellor, K. M. , Taberner, A. J. , & Loiselle, D. S. (2014). The afterload‐dependent peak efficiency of the isolated working rat heart is unaffected by streptozotocin‐induced diabetes. Cardiovascular Diabetology, 13, 4. 24387738 10.1186/1475-2840-13-4 PMC3916799

[eph13746-bib-0022] Han, J. C. , Guild, S. J. , Pham, T. , Nisbet, L. , Tran, K. , Taberner, A. J. , & Loiselle, D. S. (2018). Left‐Ventricular Energetics in Pulmonary Arterial Hypertension‐Induced Right‐Ventricular Hypertrophic Failure. Frontiers in Physiology, 8, 1115. 29375394 10.3389/fphys.2017.01115 PMC5767264

[eph13746-bib-0023] Han, J. C. , Taberner, A. , Kirton, R. S. , Nielsen, P. , Archer, R. , Kim, N. , & Loiselle, D. (2011). Radius‐dependent decline of performance in isolated cardiac muscle does not reflect inadequacy of diffusive oxygen supply. American Journal of Physiology‐Heart and Circulatory Physiology, 300(4), H1222–H1236. 21217065 10.1152/ajpheart.01157.2010

[eph13746-bib-0024] Han, J. C. , Taberner, A. J. , Loiselle, D. S. , & Tran, K. (2022). Cardiac efficiency and Starling's Law of the Heart. The Journal of Physiology, 600(19), 4265–4285. 35998082 10.1113/JP283632 PMC9826111

[eph13746-bib-0025] Han, J. C. , Taberner, A. J. , Nielsen, P. M. , & Loiselle, D. S. (2013). Interventricular comparison of the energetics of contraction of trabeculae carneae isolated from the rat heart. The Journal of Physiology, 591(3), 701–717. 23184511 10.1113/jphysiol.2012.242719 PMC3577548

[eph13746-bib-0026] Han, J. C. , Taberner, A. J. , Nielsen, P. M. , Kirton, R. S. , Ward, M. L. , & Loiselle, D. S. (2010). Energetics of stress production in isolated cardiac trabeculae from the rat. American Journal of Physiology‐Heart and Circulatory Physiology, 299(5), H1382–H1394. 20729397 10.1152/ajpheart.00454.2010

[eph13746-bib-0027] Janssen, P. M. L. , Stull, L. B. , Leppo, M. K. , Altschuld, R. A. , & Marban, E. (2003). Selective contractile dysfunction of left, not right, ventricular myocardium in the SHHF rat. American Journal of Physiology‐Heart and Circulatory Physiology, 284(3), H772–H778. 12424099 10.1152/ajpheart.01061.2001

[eph13746-bib-0028] Janssen, P. M. L. , Stull, L. B. , & Marban, E. (2002). Myofilament properties comprise the rate‐limiting step for cardiac relaxation at body temperature in the rat. American Journal of Physiology‐Heart and Circulatory Physiology, 282(2), H499–H507. 11788397 10.1152/ajpheart.00595.2001

[eph13746-bib-0029] Johnston, C. M. , Han, J. C. , Loiselle, D. , Nielsen, P. M. F. , & Taberner, A. (2016). Cardiac activation heat remains inversely dependent on temperature over the range 27–37°C. American Journal of Physiology‐Heart and Circulatory Physiology, 310(11), H1512–H1519. 27016583 10.1152/ajpheart.00903.2015

[eph13746-bib-0030] Johnston, C. M. , Han, J. C. , Ruddy, B. P. , Nielsen, P. M. F. , & Taberner, A. J. (2015). A high‐resolution thermoelectric module‐based calorimeter for measuring the energetics of isolated ventricular trabeculae at body temperature. American Journal of Physiology‐Heart and Circulatory Physiology, 309(2), H318–H324. 26001412 10.1152/ajpheart.00194.2015

[eph13746-bib-0031] Julius, S. (1988). Transition from high cardiac output to elevated vascular resistance in hypertension. American Heart Journal, 116(2 Pt 2), 600–606. 3293404 10.1016/0002-8703(88)90557-1

[eph13746-bib-0032] Kjekshus, J. K. (1986). Importance of heart rate in determining beta‐blocker efficacy in acute and long‐term acute myocardial infarction intervention trials. The American Journal of Cardiology, 57(12), 43F–49F. 10.1016/0002-9149(86)90888-x 2871745

[eph13746-bib-0033] Lamberts, R. R. , Hamdani, N. , Soekhoe, T. W. , Boontje, N. M. , Zaremba, R. , Walker, L. A. , de Tombe, P. P. , van der Velden, J. , & Stienen, G. J. (2007). Frequency‐dependent myofilament Ca^2+^ desensitization in failing rat myocardium. The Journal of Physiology, 582(Pt 2), 695–709. 17478529 10.1113/jphysiol.2007.134486 PMC2075316

[eph13746-bib-0034] Layland, J. , & Kentish, J. C. (1999). Positive force‐ and [Ca^2+^]_i_‐frequency relationships in rat ventricular trabeculae at physiological frequencies. American Journal of Physiology, 276(1), H9–18. 9887011 10.1152/ajpheart.1999.276.1.H9

[eph13746-bib-0035] Layland, J. , & Kentish, J. C. (2000). Effects of α1‐ or β‐adrenoceptor stimulation on work‐loop and isometric contractions of isolated rat cardiac trabeculae. The Journal of Physiology, 524(Pt 1), 205–219. 10747193 10.1111/j.1469-7793.2000.t01-1-00205.x PMC2269858

[eph13746-bib-0036] Layland, J. , & Kentish, J. C. (2002). Myofilament‐based relaxant effect of isoprenaline revealed during work‐loop contractions in rat cardiac trabeculae. The Journal of Physiology, 544(Pt 1), 171–182. 12356890 10.1113/jphysiol.2002.022855 PMC2290578

[eph13746-bib-0037] Layland, J. , Young, I. S. , & Altringham, J. D. (1995). The length dependence of work production in rat papillary muscles in vitro. Journal of Experimental Biology, 198(Pt 12), 2491–2499. 8576681 10.1242/jeb.198.12.2491

[eph13746-bib-0038] Litwin, S. E. , & Morgan, J. P. (1999). Effects of stimulation frequency on calcium transients in noninfarcted myocardium: Modulation by chronic captopril treatment. Journal of Cardiac Failure, 5(3), 224–235. 10496195 10.1016/s1071-9164(99)90007-6

[eph13746-bib-0039] Loiselle, D. , Han, J. C. , Mellor, K. M. , Pham, T. , Tran, K. , Goo, S. , Taberner, A. J. , & Hickey, A. J. R. (2014). Assessing the efficiency of the diabetic heart at subcellular, tissue and organ level. Journal of General Practice, 2(4), 4.

[eph13746-bib-0040] Maier, L. S. , Bers, D. M. , & Pieske, B. (2000). Differences in Ca^2+^‐handling and sarcoplasmic reticulum Ca^2+^‐content in isolated rat and rabbit myocardium. Journal of Molecular and Cellular Cardiology, 32(12), 2249–2258. 11113000 10.1006/jmcc.2000.1252

[eph13746-bib-0041] Maier, L. S. , Brandes, R. , Pieske, B. , & Bers, D. M. (1998). Effects of left ventricular hypertrophy on force and Ca^2+^ handling in isolated rat myocardium. The American Journal of Physiology, 274(4), H1361–H1370. 9575941 10.1152/ajpheart.1998.274.4.H1361

[eph13746-bib-0042] Mellor, N. G. , Pham, T. , Tran, K. , Loiselle, D. S. , Ward, M.‐L. , Taberner, A. J. , Crossman, D. J. , & Han, J. C. (2021). Disruption of transverse‐tubular network reduces energy efficiency in cardiac muscle contraction. Acta Physiologica, 231(2), e13545. 32757472 10.1111/apha.13545

[eph13746-bib-0043] Mellors, L. J. , Gibbs, C. L. , & Barclay, C. J. (2001). Comparison of the efficiency of rat papillary muscles during afterloaded isotonic contractions and contractions with sinusoidal length changes. Journal of Experimental Biology, 204(Pt 10), 1765–1774. 11316497 10.1242/jeb.204.10.1765

[eph13746-bib-0044] Milani‐Nejad, N. , Brunello, L. , Gyorke, S. , & Janssen, P. M (2014). Decrease in sarcoplasmic reticulum calcium content, not myofilament function, contributes to muscle twitch force decline in isolated cardiac trabeculae. Journal of Muscle Research and Cell Motility, 35(3–4), 225–234. 25056841 10.1007/s10974-014-9386-9 PMC4232993

[eph13746-bib-0045] Neubauer, S. (2007). The failing heart–an engine out of fuel. New England Journal of Medicine, 356(11), 1140–1151. 17360992 10.1056/NEJMra063052

[eph13746-bib-0046] Pham, T. , Han, J. C. , Taberner, A. , & Loiselle, D. (2017). Do right‐ventricular trabeculae gain energetic advantage from having a greater velocity of shortening? The Journal of Physiology, 595(20), 6477–6488. 28857176 10.1113/JP274837 PMC5638877

[eph13746-bib-0047] Pham, T. , Nisbet, L. , Taberner, A. , Loiselle, D. , & Han, J. C (2018). Pulmonary arterial hypertension reduces energy efficiency of right, but not left, rat ventricular trabeculae. The Journal of Physiology, 596(7), 1153–1166. 29363144 10.1113/JP275578 PMC5878219

[eph13746-bib-0048] Pham, T. , Tran, K. , Mellor, K. M. , Hickey, A. , Power, A. , Ward, M. L. , Taberner, A. , Han, J. C. , & Loiselle, D. (2017). Does the intercept of the heat‐stress relation provide an accurate estimate of cardiac activation heat? The Journal of Physiology, 595(14), 4725–4733. 28455843 10.1113/JP274174 PMC5509849

[eph13746-bib-0049] Pham, T. , Tran, K. , Taberner, A. J. , Loiselle, D. S. , & Han, J. C. (2022). Cross‐bridge thermodynamics in pulmonary arterial hypertensive right‐ventricular failure. Journal of Applied Physiology, 132(6), 1338–1349. 35482327 10.1152/japplphysiol.00014.2022 PMC9208464

[eph13746-bib-0050] Pham, T. , Zgierski‐Johnston, C. M. , Tran, K. , Taberner, A. J. , Loiselle, D. S. , & Han, J. C. (2019). Energy expenditure for isometric contractions of right and left ventricular trabeculae over a wide range of frequencies at body temperature. Scientific Reports, 9(1), 8841. 31222042 10.1038/s41598-019-45273-1 PMC6586799

[eph13746-bib-0051] Pieske, B. , Maier, L. S. , Bers, D. M. , & Hasenfuss, G. (1999). Ca^2+^ handling and sarcoplasmic reticulum Ca^2+^ content in isolated failing and nonfailing human myocardium. Circulation Research, 85(1), 38–46. 10400909 10.1161/01.res.85.1.38

[eph13746-bib-0052] Power, A. , Pearson, N. , Pham, T. , Cheung, C. , Phillips, A. , & Hickey, A. (2014). Uncoupling of oxidative phosphorylation and ATP synthase reversal within the hyperthermic heart. Physiological Reports, 2(9), e12138. 25263202 10.14814/phy2.12138 PMC4270237

[eph13746-bib-0053] Rouleau, J. L. , Paradis, P. , Shenasa, H. , & Juneau, C. (1986). Faster time to peak tension and velocity of shortening in right versus left ventricular trabeculae and papillary muscles of dogs. Circulation Research, 59(5), 556–561. 3802429 10.1161/01.res.59.5.556

[eph13746-bib-0054] Schouten, V. J. , & ter Keurs, H. E. (1986). The force‐frequency relationship in rat myocardium: The influence of muscle dimensions. Pflügers Archives, 407(1), 14–17. 3737379 10.1007/BF00580714

[eph13746-bib-0055] Shen, X. , Tan, Z. , Zhong, X. , Tian, Y. , Wang, X. , Yu, B. , Ramirez‐Correa, G. , Murphy, A. , Gabrielson, K. , Paolocci, N. , & Gao, W. D. (2013). Endocardial endothelium is a key determinant of force‐frequency relationship in rat ventricular myocardium. Journal of Applied Physiology, 115(3), 383–393. 23703113 10.1152/japplphysiol.01415.2012 PMC3743009

[eph13746-bib-0056] Shinke, T. , Takeuchi, M. , Takaoka, H. , & Yokoyama, M. (1999). Beneficial effects of heart rate reduction on cardiac mechanics and energetics in patients with left ventricular dysfunction. Japanese Circulation Journal, 63(12), 957–964. 10614841 10.1253/jcj.63.957

[eph13746-bib-0057] Stanford, S. C. , Gettins, D. , & Little, H. J. (1990). Adverse effects on rat cardiac function ex vivo after repeated administration of the benzodiazepine partial inverse agonist, FG7142. British Journal of Pharmacology, 99(3), 441–444. 2158841 10.1111/j.1476-5381.1990.tb12946.x PMC1917342

[eph13746-bib-0058] Taberner, A. , Nielsen, P. , Johnston, C. , Anderson, A. , Cheuk, M. , Garrett, A. , Dowrick, J. , Tang, E. L. P. , HajiRassouliha, A. , Ruddy, B. , Pham, T. , Tran, K. , Han, J. C. , & Loiselle, D. (2019). A dynamometer for nature's engines. IEEE Instrumentation & Measurement Magazine, 22, 10–16.

[eph13746-bib-0059] Taberner, A. J. , Johnston, C. M. , Pham, T. , Han, J. C. , Ruddy, B. P. , Loiselle, D. S. , & Nielsen, P. M. (2015). Measuring the mechanical efficiency of a working cardiac muscle sample at body temperature using a flow‐through calorimeter. IEEE Engineering in Medicine and Biology Society, 7966–7969. 10.1109/EMBC.2015.7320240 26738140

[eph13746-bib-0060] Taberner, A. J. , Zgierski‐Johnston, C. M. , Pham, T. , Han, J. C. , Uddin, R. , Loiselle, D. S. , Ruddy, B. P. , & Nielsen, P. M. F. (2018). A flow through infusion calorimeter for measuring muscle energetics: Design and performance. IEEE Transactions on Instrumentation and Measurement, 67(7), 1690–1699.

[eph13746-bib-0061] Tadic, M. , Cuspidi, C. , & Grassi, G. (2018). Heart rate as a predictor of cardiovascular risk. European Journal of Clinical Investigation, 48(3). 10.1111/eci.12892 29355923

[eph13746-bib-0062] Tang, L. , Gao, W. , & Taylor, P. B. (1996). Force‐frequency response in isoproterenol‐induced hypertrophied rat heart. European Journal of Pharmacology, 318(2–3), 349–356. 9016925 10.1016/s0014-2999(96)00805-9

[eph13746-bib-0063] Taylor, D. G. , Parilak, L. D. , LeWinter, M. M. , & Knot, H. J. (2004). Quantification of the rat left ventricle force and Ca^2+^‐frequency relationships: Similarities to dog and human. Cardiovascular Research, 61(1), 77–86. 14732204 10.1016/j.cardiores.2003.09.022

[eph13746-bib-0064] Triposkiadis, F. , Karayannis, G. , Giamouzis, G. , Skoularigis, J. , Louridas, G. , & Butler, J. (2009). The sympathetic nervous system in heart failure: Physiology, pathophysiology, and clinical implications. Journal of the American College of Cardiology, 54(19), 1747–1762. 19874988 10.1016/j.jacc.2009.05.015

[eph13746-bib-0065] Van Bogaert, P. P. , Goethals, M. , & Simoens, C. (1990). Use‐ and frequency‐dependent blockade by UL‐FS 49 of the if pacemaker current in sheep cardiac Purkinje fibres. European Journal of Pharmacology, 187(2), 241–256. 2272362 10.1016/0014-2999(90)90011-t

[eph13746-bib-0066] Varian, K. D. , & Janssen, P. M. (2007). Frequency‐dependent acceleration of relaxation involves decreased myofilament calcium sensitivity. American Journal of Physiology‐Heart and Circulatory Physiology, 292(5), H2212–H2219. 17209002 10.1152/ajpheart.00778.2006

[eph13746-bib-0067] Wang, E. Y. , Dixson, J. , Schiller, N. B. , & Whooley, M. A. (2017). Causes and predictors of death in patients with coronary heart disease (from the heart and soul study). American Journal of Cardiology, 119(1), 27–34. 27788932 10.1016/j.amjcard.2016.09.006

